# The Tennessee Dental Association

**Published:** 1871-10

**Authors:** 


*The Tennessee Dental Association.*—A correspondent sends us an account of the proceedings of the fifth annual meeting of this Association, which was held in Nashville, commencing on the 27th, and ending on the 29th of July last:

"During the the two days meeting peace and harmony prevailed; all present entering into the discussions upon the various subjects reported upon by the regular standing committees, such as Education, Physiology, Pathology and Surgery, Histology, Therapeutics, Operative and Mechanical Dentistry. Take the meeting altogether, it was a very good one. We are glad to say that the society has at last taken oue step towards educating the people in regard to their teeth. We will have reported at the next meeting a circular or pamphlet upon this subject. We hope the committee will succeed in getting up something that will be very interesting and instructive to the people. The next meeting will be held in Nashville commencing on the 27th of July 1872. The harmony and interest manifested in this meeting, we take as a sufficient guarantee that it will be one of the largest and most interesting we have had. We hope every good man in the State will turn out and let us have a good time generally. Officers for the ensuing year: S. C. Chisholm, President; Vice Presidents, G. C. Sandusky and J. C. Ross; Recording Sec'y; E. S. Chisholm; Corresponding Sec'y, A. Hartman; Treasurer, S. J. Cobb."

